# Effects of early and late-onset treatment with carvedilol in an experimental model of aortic regurgitation

**DOI:** 10.1186/s40064-015-0829-6

**Published:** 2015-02-01

**Authors:** Kristian Eskesen, Niels Thue Olsen, Veronica L Dimaano, Thomas Fritz-Hansen, Peter Sogaard, Theodore P Abraham

**Affiliations:** Johns Hopkins Medical Institutions, Division of Cardiology, 600 North Wolfe Street, Carnegie 568, Baltimore, MD 21287 USA; Department of Cardiology, Gentofte Hospital, University of Copenhagen, Copenhagen, Denmark; Department of Cardiology, Aalborg Hospital, University of Aalborg, Aalborg, Denmark

**Keywords:** Aortic regurgitation, Heart valvular disease, Experimental animal studies, Echocardiography, Invasive hemodynamic measurements

## Abstract

**Background:**

Beta-blockade is contraindicated in severe aortic regurgitation (AR) due to the fear of prolonging diastole and thus aggravate regurgitation. However, this has never been scientifically proven and positive effects of targeting the sympathetic system in AR has been demonstrated in several studies.

**Method:**

Thirty-nine Sprague–Dawley rats with AR were randomized to ten weeks of medical treatment with carvedilol or no treatment. Treatment was initiated either early or late after AR induction. The effect of carvedilol was assessed by serial echocardiography and invasive hemodynamic measurements.

**Results:**

AR resulted in eccentric hypertrophy and left ventricular (LV) dysfunction. LV remodeling and function as measured by echocardiography was unaffected by treatment. LV dimensions were similar between treated and untreated groups and measures of LV performance (including strain and strain rate) were also unaltered. This result was confirmed by invasive measurements showing maximal and minimal pressure–time development, LV volumes, and LV pressures, to be unaltered by treatment. On the contrary, despite relative bradycardia carvedilol did not reflect any negative impact on the heart.

**Conclusion:**

Carvedilol did not improve left ventricular remodeling or function in rats with surgically induced AR. Despite relative bradycardia, we did not find carvedilol to negatively impact the heart, either when treatment was initiated early or late in the course of disease.

## Background

Chronic aortic regurgitation (AR) is a common heart valve disease with high morbidity and mortality rates once symptoms occur (Dujardin et al. 1999). However, disease–progression is often gradual and patients with even severe degree of regurgitation may be asymptomatic for decades without any need of surgical intervention (Bonow et al. 1991; Goldbarg and Halperin 2008). Ultimately, left ventricular (LV) compensatory mechanisms are exhausted, which results in LV dilation and dysfunction and consequently heart failure and eventually death (Maurer 2006). The time of surgical intervention (aortic valve replacement/repair (AVR)) relies on when patients develop symptoms or when significant alterations in LV size or function occurs (Bonow et al. 2008; Vahanian et al. 2012). Medical treatment of AR has been debated for decades and results from clinical studies have been equivocal (Levine and Gaasch 1996; Evangelista et al. 2005; Lin and Stewart 2011). Thus, medical management of AR is not routinely recommended.

Long–term cardiac overload, as seen in congestive heart failure, leads to over–activation of the sympathetic and renin–angiotensin–aldosterone system (Schrier and Abraham 1999). This results in various deleterious effects (Levine et al. 1982; Bristow 1984) and reversal of this state has demonstrated beneficial effects on the heart (Packer et al. 1996). Beta-blockers are thus first-line treatment in congestive heart failure (Hunt et al. 2009; McMurray et al. 2012). The use of beta-blockers in AR is however controversial, mostly due to the risk of prolonging diastole, which could aggravate regurgitation. Only a few studies have addressed the effects of beta–blockade in volume overload, but the rationale for targeting the sympathetic system in AR has been shown by Champetier et al. (Champetier et al. 2009). They found several pathophysiological similarities between hearts from rats with AR and hearts exposed to other types of cardiac disease. Furthermore, in a study by Plante et al. (Plante et al. 2004) it was shown that treating AR for 24 weeks with metoprolol had a global impact on the heart, resulting in improved LV function and reduction in LV remodeling. Additionally, it has been shown in experimental and clinical studies that beta-blockers reduce mortality in chronic AR (Plante et al. 2008; Sampat et al. 2009). However, experimental studies testing the effects of beta–blockade in AR have only tested the effects of treatment started early in the course of disease. In a clinical setting, patients may have AR for years before the condition is discovered, and at this time–point LV changes may be too advanced to exploit the positive effects of beta–blockade. Thus starting treatment late may induce an increase in regurgitation–volume that could disturb the balance between load and compensatory mechanisms.

We therefore undertook this study to investigate the effects of beta-blockade in a setting reproducing a clinical situation where treatment is initiated at different time points along the course of disease.

## Results

### Clinical data

Five rats died during the study–period; one from the AR (early) group, two from the AR + CAR (early) group, one from the AR (late) group, and one from the AR + CAR (late) group. All rats dying were found dead in the morning with no preceding signs of heart failure. At sacrifice heart weight in sham (early) operated rats was significantly lower than in rats with AR (early), while no significant difference was found between AR and AR + CAR in either the early or the late treated groups, Table 1. Furthermore we did not find any differences in lung tissue weights among groups.

**Table 1:** **Morphometric data at sacrifice**

	**EARLY**					**LATE**		
	**Sham**	**AR**	**AR + CAR**	**Disease p-value**	**Treatment p-value**	**AR**	**AR + CAR**	**Treatment p-value**
	**(n = 8)**	**(n = 7)**	**(n = 7)**			**(n = 5)**	**(n = 7)**	
Rat Weight	717 ± 20	666 ± 15	698 ± 17	0,12	0,75	739 ± 54	715 ± 11	0,85
Heart weight (g)	1.66 ± 0.07	2.32 ± 0.12	2.32 ± 0.17	0.003*	1,00	2.27 ± 0.25	2.28 ± 0.15	0,98
LV weight (g)	1.17 ± 0.05	1.69 ± 0.10	1.73 ± 0.13	0.002*	0,95	1.65 ± 0.17	1.72 ± 0.11	0,75
RV weight (g)	0.26 ± 0.01	0.36 ± 0.02	0.32 ± 0.02	0.005*	0,46	0.34 ± 0.05	0.32 ± 0.02	0,70
Lung weight (g)	1.59 ± 0.03	2.00 ± 0.25	2.04 ± 0.35	0,45	0,99	1.72 ± 0.11	1.65 ± 0.05	0,60

Weights at sacrifice in sham, treated (Carvedilol), and untreated (AR) rats. Disease p-values indicate difference between sham and untreated groups. Treatment p–value indicate difference between treated and untreated groups. Left ventricle (LV), right ventricle (RV). *p < 0.05.

### Echocardiographic data

**Figure 1:**
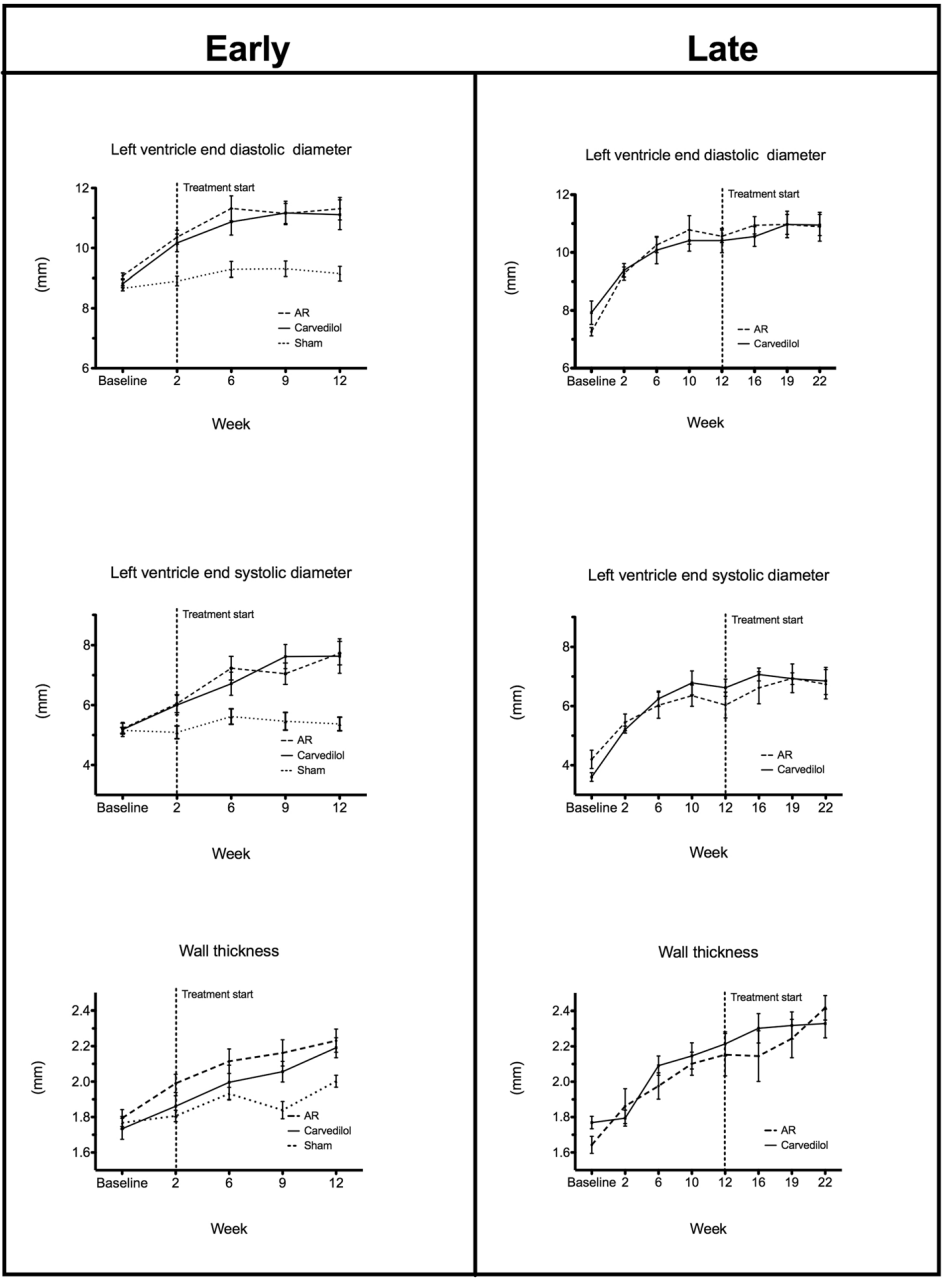
**LV dimensions.** LV dimensions measured by serial echocardiography in sham-operated (sham), treated (Carvedilol), and untreated (AR) in the early (left) and late (right) treated groups.

**Figure 2:**
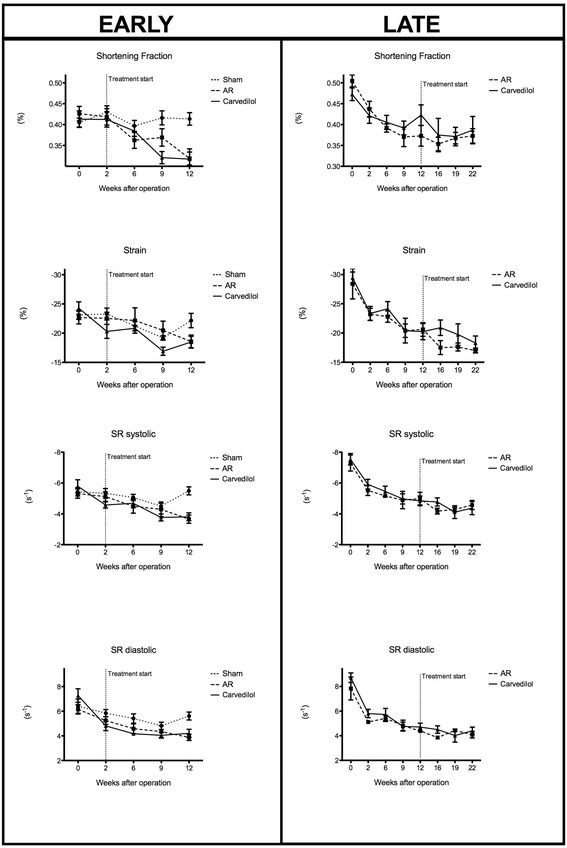
**LV function.** LV function measured by different echocardiographic techniques in sham-operated (sham), treated (Carvedilol), and untreated (AR) in the early (left) and late (right) treated groups. Strain rate (SR).

At baseline no echocardiographic differences were observed between groups. Rats with AR developed eccentric hypertrophy with increased LV dimensions (LVEDD and LVESD) and wall thickness, Table 2. Additionally, AR resulted in LV dysfunction. Carvedilol had no effect on this, and echocardiographic measures of LV remodelling was unaffected by treatment in both the early and late treated group, Figure 1. Also the rate of LV systolic dysfunction–development measured by conventional or speckle-tracking echocardiography was unaltered by treatment in both groups, Figure 2.

**Table 2:** **Echocardiographic data**

	**EARLY**					**LATE**		
	**Sham**	**AR**	**AR + CAR**	**Disease p-value**	**Treatment p-value**	**AR**	**AR + CAR**	**Treatment p-value**
	**(n = 8)**	**(n = 7)**	**(n = 7)**			**(n = 5)**	**(n = 7)**	
LVIDd (mm)	9.1 ± 0.24	11.3 ± 0.37	11.1 ± 0.50	0.001*	0,92	10.9 ± 0.50	11.0 ± 0.36	0,92
LVIDs (mm)	5.4 ± 0.23	7.7 ± 0.39	7.6 ± 0.57	0.001*	0,99	6.9 ± 0.46	6.7 ± 0.50	0,88
Wall thickness (mm)	2.0 ± 0.03	2.2 ± 0.07	2.2 ± 0.06	0.019*	0,87	2.4 ± 0.07	2.3 ± 0.08	0,45
FS (%)	41.4 ± 1.50	31.9 ± 1.53	31.70 ± 2.47	0.001*	1,00	37.3 ± 0.02	38.7 ± 0.03	0,75
Strain (%)	−22.15 ± 1.23	−18.5 ± 1.12	−18.5 ± 0.98	0,075	1,00	−17.0 ± 0.38	−18.1 ± 1.16	0,49

Echocardiographic data after twelve (left) and twenty-two (right) weeks of severe AR. Disease p-values indicate difference between sham and untreated (AR) groups. Treatment p-value indicates difference between treated (Carvedilol) and untreated (AR). All p-values are from paired analysis. Left ventricle end-diastolic diameter (LVEDD), left ventricle end-systolic diameter (LVESD), fractional shortening (FS), and wall thickness (WT). *p < 0.05.

Thus, we did not find carvedilol to alter the development of eccentric remodeling or LV dysfunction as measured by echocardiography, regardless of treatment–start.

### Hemodynamic measurements

Invasive hemodynamic measurements were performed at baseline and repeated immediately before sacrifice. Although only borderline significant in the late treated group we observed a lower heart rate in carvedilol–treated compared to untreated rats.

Carvedilol did not result in any significant improvement in LV maximum or minimum pressure–time development (dp/dt_max_ and dp/dt_min_) or LV volumes (relative end diastolic or end systolic volume) compared to untreated rats. Furthermore, end–diastolic and end–systolic LV pressures were unaltered by treatment in both study-legs, Table 3. We found tau to be lower in treated compared to untreated rats in the late treated group, while other measures of LV diastolic function was unchanged by treatment. It may reflect less ECM disturbance in the treated group, but this is an isolated finding and should be interpreted in the scope of a low sample size.

**Table 3:** **Invasive hemodynamics**

**Panel a**		**Early**		
	**Sham**	**AR**	**AR + CAR**	**p-value**
	**(n = 6)**	**(n = 6)**	**(n = 5)**	
HR	355 ± 11	324 ± 7	305 ± 20	0.04*
ESP (mmHg)	120 ± 7.73	124 ± 9.45	136 ± 8.28	0,75
EDP (mmHg)	6.3 ± 1.7	4.9 ± 1.3	8.2 ± 2.1	0,87
ESV (arbitrary units)	18.8 ± 0.85	23.1 ± 1.14	20.8 ± 0.99	0,55
EDV (arbitrary units)	21.2 ± 0.89	26.1 ± 1.56	22.8 ± 1.1	0,39
Tau (msec)	12.5 ± 0.90	11.5 ± 0.88	13.8 ± 1.12	0,67
dp/dt maximum (mmHg/sec)	7419 ± 292	7284 ± 303	7831 ± 333	0,81
dp/dt minimum (mmHg/sec)	−7139 ± 314	−5317 ± 376	−6351.6 ± 765	0,42
**Panel b**		**LATE**		
	**AR**	**AR + CAR**	**p-value**	
	**(n = 4)**	**(n = 7)**		
HR	368 ± 14	333 ± 12	0.09	
ESP (mmHg)	148 ± 4.1	149 ± 10.0	0,35	
EDP (mmHg)	7.2 ± 0.50	12.6 ± 0.57	0,16	
ESV (arbitrary units)	21.3 ± 0.85	20.7 ± 0.47	0,26	
EDV (arbitrary units)	23.7 ± 0.78	22.6 ± 1.37	0,86	
Tau (msec)	18.2 ± 1.47	13.2 ± 0.99	0.03*	
dp/dt maximum (mmHg/sec)	6473 ± 386	7770 ± 443	0,52	
dp/dt minimum (mmHg/sec)	−7120 ± 1473	−6635 ± 594	0,54	
SBP (mmHg)	131 ± 5.6	146 ± 7.7	0,36	
DBP (mmHg)	65.0 ± 3.3	70.3 ± 5.5	0,26	

Invasive hemodynamic measurements at sacrifice in the early (panel a) and late treated (panel b) groups. P-values are from paired analysis comparing treated (Carvedilol) and untreated (AR) groups. Pressure-volume time development (dp/dt), end-systolic pressure (ESP), end-diastolic pressure (EDP), end-systolic volume (ESV), end-diastolic volume (EDV), systolic blood pressure (SBP), and diastolic blood pressure (DBP). *p < 0.05.

Overall, we did not observe carvedilol to consistently or significantly change LV hemodynamic conditions, either when treatment was started early or late in the course of disease.

## Discussion

In this study of rats with surgically induced AR we demonstrate the cardiac effects of carvedilol when treatment is initiated early and late in the course of disease. Rats with AR clearly developed eccentric remodeling. Treatment with carvedilol did not change this. Thus, we found LV–function and remodeling, as measured by echocardiography and invasive hemodynamic catheterization to be unaltered by ten weeks of carvedilol treatment. Carvedilol induced relative bradycardia without revealing negative impact on the heart.

In a clinical setting AVR is indicated in patients with chronic severe AR once symptoms or significant alterations in LV size or function occurs (Vahanian et al. 2007; Bonow et al. 2008). AVR is an effective treatment with good long-term prognosis if performed at the correct time (Klodas et al. 1997). However, open chest surgery and anticoagulant treatment carries a considerable peri–operative risk. Consequently, medical therapy to alleviate LV load and ultimately postpone the time of surgery has long been sought (Lin and Stewart 2011).

### Blockade of the sympathetic system

In heart failure it is known that activation of neurohormonal systems exerts deleterious effects on the heart (Bristow 1984). Additionally, when chronically increased, high concentrations of circulating catecholamines are cardiotoxic and the extra inotropic and chronotropic drive results in excessive oxygen expenditure (Mann et al. 1992). Blocking this over–activation can reverse these negative effects. Nevertheless, due to the risk of bradycardia and the following increase in regurgitation and afterload, beta-blockers are contraindicated in severe AR. This relies on little scientific proof and the time is right to investigate this further. Recently it was shown that sustained volume overload activate neurohormonal systems, similar to other types of cardiac disease, and genetic studies has proven beta–blockade to reverse up–regulated genes related to this over–activation (Suzuki et al. 1997; Champetier et al. 2009; Zendaoui et al. 2011). In our study the aim of treatment was to only bluntly inhibit the sympathetic system, as to reduce the risk of negative consequences on the heart while still exploiting the possible positive biological properties of carvedilol. This was achieved by a small dose of carvedilol to only mildly reduced HR. In previous studies of AR a similar small dose of beta-blockers has been used, but has mainly investigating the effects of early-started treatment (Suzuki et al. 1997; Plante et al. 2004; Zendaoui et al. 2011). Treatment was well tolerated in both (early and late started) groups and we did not observe any reflections of negative consequences on hemodynamic and morphometric measurements, as would be expected if regurgitation was significantly increased (e.g. cardiac incompensation).

### LV remodeling and function

Severe AR is initially characterized by a long compensated phase, where hemodynamic consequences of sustained volume overload causes an adaptive increase in LV volumes without reduction in LV performance. Eccentric hypertrophy develops to compensate the initial overload–burden, but once compensatory mechanisms are exhausted, more ventricular load results in further remodeling and increased afterload. In this process, loss of elastin and alterations in collagen fiber organization results in decreased LV compliance and increased stiffness. Gradually filling pressures and cardiac work increases that ultimately result in decreased cardiac performance.

Similar to the natural history in man, we observed AR rats to develop LV remodeling. This process started early after AR-induction and continued throughout the course of disease. This confirms the findings of Plante et al. (Champetier et al. 2009) who found in a model of experimental AR that sarcomeric alpha–actin and smooth muscle alpha–actin protein were significantly increased along with increased levels of mRNA for several ECM component, including collagen I and III. This was reflected on LV dilation and increased relative wall thickness measured by echocardiography. In their study early started metoprolol reduced LV end systolic diameter but did not change wall thickness. This was explained by a change in several ECM components rather than reversal of myocyte proteins. Similarly, in a study by Zendaoui et al. (Zendaoui et al. 2011), a high–dose beta–blocker initiated early has shown comparable effects. Thus, they found six months of treatment to reduce end–systolic diameter and expression of mRNA for collagen I, while echocardiographic measures of hypertrophy was unaffected. We found a similar result of unchanged hypertrophy in both early and late treated groups, although we found no difference in LV diameters. This could be explained by differences in AR severity and heart rate response amongst studies but more likely due to long-term versus short-term treatment regimes. As an isolated result we found (tau) to be decreased in the late treated group, which could reflect less ECM disturbance. In conclusion these results indicate that beta–blockade may positively influence LV dimensions but has not shown to be able to consistently reduce myocyte hypertrophy.

We did not find any difference in positive or negative dp/dt and LV filling pressures were similar between sham–operated and rats with AR in both study–legs. Treatment did not affect this. These results partly confirm the finding of others.

Previous studies have shown inconsistent effects on LV performance. In one study, long–term treatment with metoprolol did not change pressure development or LVEF after 6 and 12 months (Plante et al. 2008), while another study showed dp/dt_min_ to be significantly increased by disease and dp/dt_max_ to be unaffected, while high–dose carvedilol did not change this. In the same study LVEF measured by echocardiography decreased marginally in rats with AR (LVEF 77 ± 0.8% in sham vs. 66 ± 0.5% in AR) and carvedilol slightly reversed this mild decrease (71 ± 1.4% in treated vs. 66 ± 0.5% in untreated rats). In another study invasive hemodynamic measurements were not performed but echocardiography showed improved systolic and diastolic function in rats treated early with metoprolol for twenty–four weeks. However, in this study LV systolic function was also only marginally affected by treatment. We did not find carvedilol to improve LV systolic function. In the studies showing positive effects systolic function, LVEF was within the limits of reference and did not decrease significantly during the course of disease. This may reflect a more benign type of AR where six months of severe volume overload does not impair systolic function. Our model somehow resulted in a reduction in LV function measured by FS and speckle tracking echocardiography and may represent a more progressive type of disease less susceptible to the positive effects of beta–blockade.

Although we were unable to show carvedilol to improve LV remodeling and performance, regardless of treatment start, we did not find beta–blokade to negatively impact the heart.

### Limitations

For several reasons (e.g. great biological differences) animal studies cannot and should never be extrapolated to humans.

Using a higher dose of carvedilol or a different beta-blocker (e.g. a selective) may show more pronounced results. However, we sought to only bluntly inhibit the sympathetic system in fear of increasing regurgitation. The dose of carvedilol chosen resulted in a heart rate drop of 5-10% compared to non-treated AR groups. This is similar to what has previously shown significant effects on the heart on other experimental studies. Although selective and non-selective beta-blockers have pharmacological actions in common they differ greatly, and cannot be directly compared. Thus, we interpret our results in the scope hereof.

Due to a small sample size it is hard to conclude definitively on our results and thus we suggest a larger scale study to investigated this interesting field. However, we believe that we have interpreted our findings accordingly.

## Conclusion

Carvedilol did not improve left ventricular remodeling or performance in rats with surgically induced severe AR. Despite relative bradycardia, we did not find carvedilol to negatively impact the heart, either when treatment was initiated early or late in the course of disease.

## Methods

### Animal model

AR was created in rats by echocardiography–guided closed–chest operation, as described elsewhere (Arsenault et al. 2002). Briefly, two aortic valve leaflets were punctured in a retrograde manner by a right–sided carotid arteriotomy. Isoflurane (1.5-2.0% mixed with oxygen) sedation was followed by an intra peritoneal cocktail (ketamine 90 mg kg^−1^ and Xylazine 10 mg kg^−1^) injection. AR perforation and severity was confirmed by echocardiography during the procedure. Treatment was performed by mixing carvedilol (10 mg/kg/d) in transgenic dough–diet (Bioserv: Frenchtown, NJ, U.S.A.), which was fed to rats daily for the duration of medical treatment. All animal experiments were approved by the Johns Hopkins University Institutional Animal Care and Use Committee.

#### Early treatment protocol

The twenty-five male Sprague Dawley rats (age 19–20 weeks: Charles River: Wilmington, MA, U.S.A.) included in this part of the study were divided into three groups: Sham–operated rats receiving no treatment (Sham (early), n = 8), rats with AR receiving no medical treatment (AR (early), n = 8), and rats treated with carvedilol beginning two weeks after AR induction (AR + CAR (early), n = 9). In sham–operated rats all procedures were performed, except perforation of aortic valves. All rats were sacrificed twelve weeks after induction of AR.

#### Late treatment protocol

Fourteen male Sprague Dawley rats (age 9–10 weeks) were subjected to severe AR and divided into two groups: Controls with severe AR receiving no medical treatment (AR (late), n = 6) and rats with severe AR treated with carvedilol beginning at twelve weeks after induction of AR (AR + CAR (late), n = 8). All rats were sacrificed twenty–two weeks after induction of AR.

### Echocardiography

Echocardiography was performed on a Vivid 7 machine (GE Healthcare: Horton, Norway) with a 14 MHZ linear vascular probe in unconscious rats anesthetized with gas (Isoflurane 1.5–2.0%). Examinations were performed at following time points: *Early study*: 0 (baseline), 2, 6, 9 and 12 weeks after AR induction. *Late study*: 0 (baseline), 2, 6, 9, 10, 12, 16, 19, 22 weeks after AR induction. Regurgitation was confirmed by a color-Doppler ratio of regurgitant jet >50% of the LV outflow tract diameter. LV measurements were assessed by conventional M-mode echocardiography; end diastolic diameter (LVEDD), end systolic diameter (LVESD), shortening fraction (FS) and wall thickness (WT). Measurements were averaged from four beats, two parasternal long–axis (PLAX) and two short–axis (SAX) planes. FS was calculated as [LVEDD–LVESD]/ [LVEDD]. As a measure of WT, septum and LV posterior wall were averaged. Speckle tracking was performed by acquiring 2D cine loops from at least six cardiac cycles, in mid–ventricular SAX plane at frame rates >70 s^−1^. Tracking was performed by using at least three consecutive cardiac cycles in EchoPAC’s strain modality analysis software (v. 7.0, GE Healthcare, Waukesha, WI, U.S.A.). Averaged circumferential deformation represented by the global trace was used. For strain–rate measurements the global traces were used.

### Hemodynamic measurements

A micro–tip conductance pressure–volume catheter (Millar Instruments, Inc.: Houston, TX, U.S.A.) was inserted into the heart by right–sided carotid arteriotomy using a classic closed–chest approach (Pacher et al. 2008). Pressure-volume (PV) loops were obtained under resting conditions. Measurements were performed at baseline and immediately before sacrifice.

### Statistical analysis

By comparing sham and untreated rats the overload caused by AR was documented. The effect of medical treatment was documented by comparing untreated AR-rats with carvedilol-treated AR-rats. The difference between multiple groups was analyzed with ANOVA and Tukey’s test for multiple comparisons. Serial measurements were compared by repeated measures. All p–values are two–tailed and a significance level of 0.05 was used. Statistics are given as mean ± SEM, unless stated otherwise. All analysis were performed using SAS® software (SAS for windows, release 9.1, SAS Institute Inc., Cary, NC, U.S.A.) and PASW statistics 18.0 (SPSS IBM for Macintosh, release 18.0.3, Armonk, USA).

## Abbreviations

AR: Aortic regurgitation
AVR: Aortic valve replacement/repair
DBP: Diastolic blood pressure
ECM: Extra cellular matrix
EDD: End diastolic diameter
EDP: End-diastolic pressure
EDV: End-diastolic volume
ESD: End systolic diameter
ESP: End-systolic pressure
ESV: End-systolic volume
HR: Heart rate
LV: Left ventricle
PLAX: Parasternal long–axis
PV: Pressure-volume
dp/dt: Pressure-volume time development
RV: Right ventricle
SAX: Short–axis
FS: Shortening fraction
SR: Strain rate
SBP: Systolic blood pressure
WT: Wall thickness
